# Analysing the Relation between Passion, Motivation, and Subjective Well-Being in Sport: A Systematic Review

**DOI:** 10.3390/sports12100279

**Published:** 2024-10-16

**Authors:** Teresa Bento, Anabela Vitorino, Luís Cid, Diogo Monteiro, Nuno Couto

**Affiliations:** 1 Sport Sciences School of Rio Maior, Santarém Polytechnic University (ESDRM-IPSantarém), 2040-413 Rio Maior, Portugal; teresabento@esdrm.ipsantarem.pt (T.B.); anabelav@esdrm.ipsantarem.pt (A.V.); luiscid@esdrm.ipsantarem.pt (L.C.); 2 Research Center in Sport Sciences, Health Sciences and Human Development (CIDESD), 5001-801 Vila Real, Portugal; diogo.monteiro@ipleiria.pt; 3 School of Education and Social Sciences (ESECS), Polytechnic of Leiria, 2411-901 Leiria, Portugal

**Keywords:** passion, motivation, behavioural regulation, subjective well-being, sports

## Abstract

Both the Dualistic Model of Passion and Self-Determination Theory suggest that the persistence of a behaviour over time derives from the internalisation of the passion or motivation that the individual feels about the activity. However, the integration of these theoretical assumptions may lead to a better understanding of related outcomes, in particular on subjective well-being (SWB). In this context, this study aimed at systematically reviewing the relation between passion, behaviour regulation (i.e., self-determined motivation), and SWB in sport. The PRISMA 2020 protocol was used to guide the systematic review. Electronic searches were conducted in the Web of Science database and Pubmed. The following descriptors were used: SWB; passion; motivation; behavioural regulation; and sport. After the search, 12 studies were retrieved for analysis that show us the following results tendencies: harmonious passion (HP) and autonomous motivation (AM) (i.e., more self-determined forms of behaviour regulation) are positively related to SWB (i.e., life satisfaction and positive affect), while obsessive passion (OP) and controlled motivation (CM) (i.e., less self-determined forms of behaviour regulation) are negatively associated with SWB. However, no studies were found to simultaneously analyse the three theoretical constructs. These results show us the importance of developing conditions in sport contexts that promote positive feelings that take into account athletes’ development of harmonious passion and self-determined behaviour regulation, in order to achieve higher levels of well-being. Nevertheless, these results also lead us to the necessity for more studies to highlight the theoretical link between passion and motivation in the context of sport, especially regarding the mediating role of motivation in the relationship between passion and well-being.

## 1. Introduction

Subjective well-being (SWB) is considered a long-term state with the presence of positive and absence of negative affect and life satisfaction [1,2]. It is considered to be the personal assessment that people make of their lives, judging their quality of life through feelings including states of mood and emotions, functioning as a positive factor for people’s health and longevity [1]. Understanding SWB is essential for creating and maintaining health and productive societies [3]. In this way, sports participation has been positively linked to SWB [4]. It contributes to increasing self-esteem, self-efficacy, positive self-concept, and body image and reducing psychological and physiological stress, leading to feelings of satisfaction and happiness [5]. 

However, there are some situations in the sports participation domain that can negatively influence well-being. Athletes make numerous sacrifices to improve performance [6,7] and can encounter a range of stressors related to their sport, such as overtraining, injuries, performance struggles, career changes, and the high expectations of coaches, teammates, and the public [8]. The expectations placed on athletes may be unrealistic and may have negative consequences on well-being [9]. The fear of failure in this population is related to burnout and psychological stress [10]. Effectively, factors related to both sporting factors and non-sporting factors can compromise mental health and well-being [8]. These emphasise the need to understand the motivational determinants that influence the athlete’s behavioural outcomes and well-being.

Self-Determination Theory (SDT) [11], which explains how individuals regulate their behaviour, offers a robust framework for examining how motivation affects psychological and behavioural outcomes and has been widely applied in various areas (e.g., sport, exercise, education, health, and work) [12,13,14,15,16]. In general, SDT is an approach to human motivation that considers the personality factors, as well as the causes and consequences of self-determined behaviours, disconnecting from the intrinsic versus extrinsic dichotomy, adopting, as suggested by the organismic theory [17], a motivational continuum that varies between the concepts of autonomous/self-determined motivation (i.e., identified, integrated, and intrinsic) and controlled/non self-determined motivation (i.e., amotivation, external, and introjected). According to its authors [11,17,18,19,20], this theory tells us that behaviour regulation is not directly related to the factors of social involvement, since the influence of these (e.g., training climate and coaches’ behaviour) is mediated by the satisfaction of three basic psychological needs (BPNs), competence, autonomy, and relatedness [20] (p. 13). Its satisfaction can lead to increased enjoyment, persistence, life satisfaction, and positive affect, while its frustration results in boredom, dropout, decrease in life satisfaction, and negative affect [11,17,18,19,20] 

According to Ryan and Deci [17], there is a link between the way people regulate their behaviour and SWB, since people who regulate motivation autonomously demonstrate greater positive affect and greater satisfaction with life (SWL), while people who regulate their behaviour according to controlled motivation are more likely to experience negative affect, reduce life satisfaction, and psychological distress. The motivational determinants have great impact on sport persistence or dropout [21,22,23], and for that reason, fostering autonomous motivation can be a strategy for promoting SWB among athletes. 

In addition, the Dualistic Model of Passion (DMP) [24] give us insight into how passion influences behavioural regulation and psychological outcomes in sports. According to this model, Harmonious Passion (HP) and Obsessive Passion (OP) represent two distinct forms of passion and have differentiated implications in terms of behavioural regulation. HP is associated with an autonomous regulation, because the subject practises the sporting modality by free will, due to the taste he has for the activity and not for questions of strengthening his personal identity, and, on the other hand, OP is linked to a controlling regulation because the subject forces the practice of the modality, seeking, through its realisation, feelings of social acceptance or of increased self-esteem, pressing himself internally to realise it [25]. Empirical evidence has shown that HP and OP developed different psychological outcomes since HP is more associated with positive experiences and OP with negative experiences [25,26]. In the domain of sport, several studies pointed to a strong relationship between HP and life satisfaction (e.g., Teixeira et al. [27]), affect (e.g., Verner-Filion et al. [28]), and also better performance (e.g., Cid et al. [29], Vallerand et al. [30]). 

Regarding the theoretical models, a set of revisions have been made in relation to the SDT, both in the context of the exercise [31] and in the sport context [32,33], that have sought to relate the SDT to different variables. However, a reduced number of review studies on the DMP were identified, especially regarding studies that integrated both theoretical models. One of the reviews, made by the team from which the theory of the DMP originated [34], focuses on the studies this set of researchers developed over time and analyses the relationship between passion and various areas of the individual’s life (intra-personal, interpersonal, and intergroup and in their relationship with the surrounding environment) and aims to support the DMP theory itself. Another more recent review by Curran et al. [35] aimed at analysing the relationship between Vallerand’s two passions (harmonious and obsessive) and intrapersonal outcomes and testing the moderating role of age, gender, domain, and culture. 

Despite the research on this field leading to strong evidence of the role of passion [36] and motivation [17] on well-being, there remains a notable gap in the understanding of the empirical relationship between the DMP, SDT, and SWB, especially in the sport domain. While previous reviews have focused on the individual theories of the DMP [34] and SDT, limited attention has been given to the effect of their combination on athletes’ SWB. 

Both the DMP and SDT suggests that the persistence of a behaviour over time derives from the internalisation of the passion or motivation that the individual feels about the activity. However, the integration of these theoretical assumptions may lead to a better understanding of related outcomes, in particular with regard to SWB. Although previous research suggested positive associations between these variables (e.g., Vallerand et al. [37], Rodrigues et al. [38]), this area remains understudied in the competitive sports context.

For these reasons, considering the impact that sports can have on SWB and the relationship between the DMP and SDT and SWB and to further the knowledge in the context of sport, this study aims at systematically reviewing the relation between passion, behaviour regulation, and SWB in sport. 

This paper’s purpose is to contribute to our understanding of the psychological foundations underlying a key aspect of athlete well-being and to provide some evidence-based guidelines, grounded in psychological theory and research, for how athlete SWB and can be supported within sports settings.

## 2. Materials and Methods

The PRISMA 2020 protocol was used as a reference for the preparation of this systematic review [39].

### 2.1. Selection Criteria

In the selection of the studies, the following inclusion criteria were considered: (a) samples of male and/or female sex with an age of 14 years or more. This criterion is related to the ability to understand the concept of “passion” that, according to Vallerand [40], only individuals in this age group have the ability to understand. (b) Samples of individuals apparently healthy and without indication of disability or disease; (c) publication languages being Portuguese, Spanish, or English. This criterion relates to the variety of studies that may be published in these languages. (d) Analysis of the relationship between passion and motivation and well-being; (e) framing of the studies and their analyses in the Theory of Self-Determination (SDT) and/or the Dualistic Model of Passion (DMP) with the subjective well-being model (SWB); (f) sample collection context and characteristics are that of sport; and (g) variables in the study were evaluated through the following measures: passion evaluated with the Passion Scale (PS) from Vallerand et al. [24]; motivation with the Sport Motivation Scale—version 1 or 2 (SMS)—from Pelletier et al. [41] or the Behavioural Regulation in Sport Questionnaire (BRSQ) from Lonsdale et al. [42]; SWB assessed with the Satisfaction With Life Scale (SWLS) from Diener et al. [43] and with the Positive and Negative Affect Schedule (PANAS) from Watson et al. [44]. These scales were chosen because they are the most widely used in the context of sports and are the most suited to the theoretical framework chosen in this study.

The exclusion criteria used were as follows: (a) studies that are not framed in the theories mentioned above; (b) the study objective is not the analysis of the relationships of the concepts indicated earlier; (c) the study does not include relevant data; (d) the study was not carried out with humans; (e) the study contexts are those of physical activity, exercise, leisure, education, work, or therapeutic intervention; or (f) the study evaluated the variables through other instruments than those considered in the inclusion criteria.

Intervention studies, validation studies, randomised controlled trials, clinical studies, systematic reviews, meta-analysis, or other studies were included in the sample if they met the inclusion criteria or contained relevant data.

### 2.2. Sources of Information, Research, and Data Extraction

For this systematic review, research was carried out by means of searches in the electronic databases Web of Science (WOS) and Pubmed during January of 2024 considering the period from 1985 on (date of publication of the theory of Self-determination) and using the descriptors “well-being” (wellbeing; bien-estar; bem-estar); “subjective well-being” (subjective wellbeing; bien estar subjectivo; bem-estar subjetivo); “passion” (pasióon; paixão); behavioural regulation (regulación conductal; regulação comportamental); “behaviour motivation” (regulación de la motivación; regulação da motivação), and “sport” (deporte; desporto). The descriptors were used in isolation or in combination with the bolean operators “and” or “or” (“e”; “ou”), depending on the level of search and according to the specific operation of each database. All databases were searched in Web of Science and Database Pubmed Central was used in searches in Pubmed. Filters for the following were applied: age and studies conducted with humans. Additionally, the reference lists of the studies were searched, and the authors were asked for the full articles that were not possible to obtain by other means of search. The data extracted from studies included the following: author, year, country, sample size, age/characteristics, design, objectives, evaluated variables, measurement instruments, results, statistical analysis/observations, conclusions, and the theoretical model used. 

### 2.3. Quality and Risk of Bias Assessment 

Two different tools were used to assess the risk of bias of studies included in the present review, according to study design and following the indications by Ma et al. [45]. The Quality Assessment Tool for Observational Cohort and Cross-Sectional Studies [45] includes 14 criteria related to the following: the research question, study population, groups recruited from the same population and uniform eligibility criteria, sample size justification, exposure assessed prior to outcome of measurement, sufficient timeframe to observe an effect, different levels of the exposure effect, exposures measurement, repeated exposure assessment, outcomes measurement, blinding of outcomes assessors, follow-up rate, and statistical analysis. A global assessment of the publication was undertaken, and each article was categorised as good, fair, or poor.

The Quality Assessment Tool for before and after (pre–post) studies with no control group [45] includes 12 criteria related to the following: study purpose, inclusion criteria and population, eligibility of participants, sample size, description of intervention, data quality regarding dependent variables, blinding process, follow-up rates, data analysis, multiple outcome measures, inter- and intra-individual variability, and a global assessment (good, fair, or poor). For each study, the appropriate tool was chosen according to its study design. 

## 3. Results

**Figure 1:**
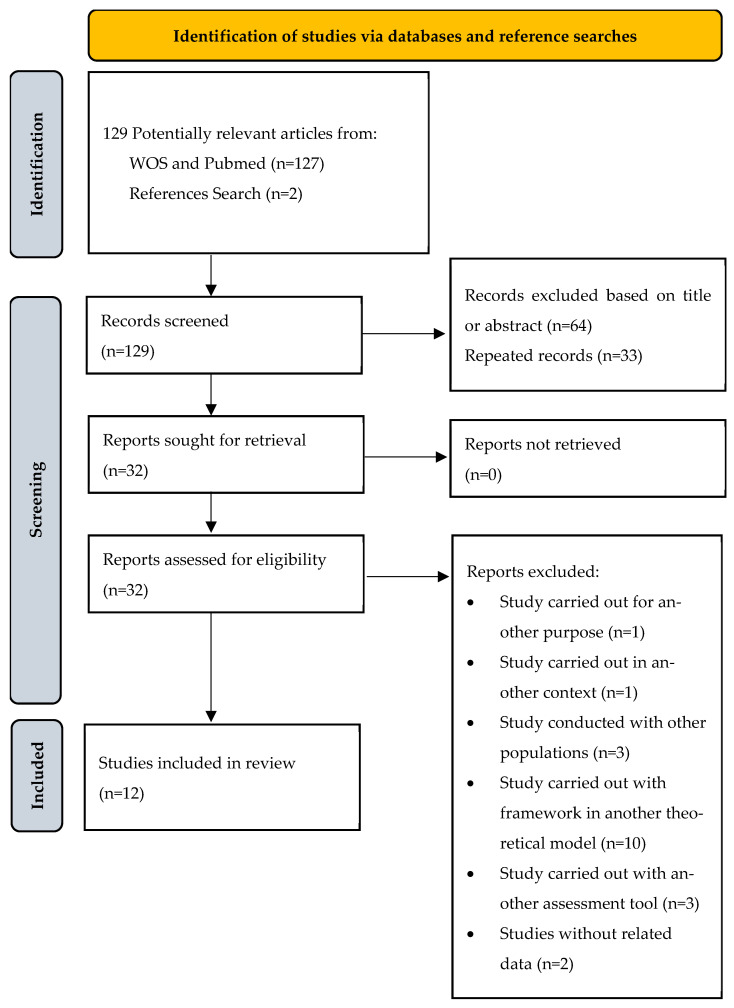
PRISMA flowchart of article inclusion, interest, or demonstration that the sample size was sufficiently large to provide confidence in the findings. Nonetheless, in the combination of all criteria, all pre–post studies were rated as good.

The initial search in the databases with the combination of the keywords well-being; passion; motivation; and sport identified 1047 hits, of which 129 potentially relevant studies were identified considering the analysis of the titles (Figure 1). After the analysis of the abstracts, 32 articles were selected according to the pre-defined inclusion and exclusion criteria. After reading the full texts, 12 studies were selected to be included in the final sample, with most of the studies being cross-sectional (62.5%), and those that presented a longitudinal design ranged from four weeks to a sports season.

Regarding the selected studies’ characteristics (Table 1), they integrate athletes of various modalities: athletics, badminton, basketball, skiing, football, gymnastics, weightlifting, hockey, ice hockey, Greco-Roman wrestling, swimming, synchronised swim, netball, water pole, volleyball, and taekwondo. There is one study that refers to individual and group sports without identifying modalities [46], another one indicates the number of sports (18) without discriminating them [47], and yet another study which indicates “various” [48]. Similarly, there is a study that includes athletes and coaches [49].

All articles were published between 2003 and 2017 and are mainly from Canada [30,49,50,51] and the United Kingdom [46,52].

The included articles included a total of 4143 individuals. The studies´ sample size ranged from 33 to 609 individuals, and the age ranged between 7 and 46 years, although the highest incidence was between the ages of 15 and 30. In the study that also included coaches, ages ranged from 18 to 72 years [49]. As an exception, two studies with an average age of under 14 years were included in the sample, based on the representativity of the articles and on the fact that they did not evaluate passion [47,53].

Most of the analysed studies include individuals of both sexes, although studies exist exclusively with females (e.g., gymnastics) or males (e.g., football players).

Studies, in general, analyse the relationship between passion (harmonious and obsessive) and well-being. Some studies discuss the role of BPNs and well-being, while others analyse the relationship between motivation and well-being. No studies were found to simultaneously analyse the three constructs.

**Table 1:** Characteristics of the included studies.

Author/Year	Country	Sample	Design	Study Goals	Outcomes	Conclusions	Risk of Bias
Verner-Filion et al., 2017 [28]	-	Study 1: 172 football athletes (122 M; 50 F); AM = 14.61 ± 1.63 y.	Study 1: Transversal (in an indoor football tournament at the provincial level, before half the season)	Study 1: Analyse the role of mediation of BPN satisfaction and deliberate practice between HP, OP, and performance in football players.	Study 1: BPNs; deliberate practice; HP; OP; SWL; performance in football players.	Study 1: HP is a positive and significant predictor of both BPN satisfaction and SWL; OP is a negative but not significant predictor of SWL; BPN satisfaction is a positive and significant predictor of SWL.	G
	-	Study 2: 598 hockey athletes (598 males).	Study 2: Longitudinal (training field of the 2000–2001 season, after practice)	Study 2: Analyse the mediating role of AGs between the P (H and O) and the BPNs, SWL, deliberate practice, and performance in hockey players.	Study 2: HP and OP; BPNs; SWL; deliberate practice; performance; AG.	Study 2: HP was associated with both types of goals (i.e., mastery and performance) that, consequently, lead to the satisfaction of BPNs, higher levels of SWL, deliberate practice, and performance. OP was associated with performance goals (i.e., approach and avoidance). This association with performance-approach and performance-avoidance goals allows for a more detailed understanding of the ambiguous relationship OP holds with BPNs, SWL, and performance. Performance-approach goals mediate the positive relation between OP and all these outcomes, performance-avoidance goals also act as a mediating variable in the negative relation between these variables.	
Carpentier and Mageau (2013) [49]	Canada	340 athletes; ages: 11–35 y (AM = 15.21 y); 13 sports (synchronised swimming, football, athletics, ice hockey, etc.); 58 coaches (48% M; 52% F); age: 18–72 y (AM = 31.14 y).	Transversal	Study the impact of change-oriented feedback on its two functions (motivating athletes and guidance for performance improvement) and the impact at the SWB level.	Athletes: PA and NA; SWL; regulation of motivation. BPNs; SV; SE.	More self-determined motivation is a positive predictor of SWB, BPNs, SV, and SE; less self-determined motivation is a negative predictor of SWL, SV, and SE; The motivated regulation is linked with reduce perception of BPNs, SWB, and SE.	G
Felton and Jowett (2013) [46]	United Kingdom	430 athletes (166 M; 264 F); age: 15–35 y (AM = 20.4 y); 59% individual sports; 41% group sports.	Transversal	Examine whether BPN satisfaction is a mechanism by which athletes’ attachment styles are associated with WB levels.	Attachment style; BPNs (parents and coaches); SV; SE; PA; and NA.	BPNs, both in parents and coaches, were positive and significant predictors of SV and PA; BPNs are a negative and significant predictor of NA; BPNs were a positive and significant predictor of SE in parents and a positive but not significant predictor of SE regarding coaches.	G
López-Walle et al., (2012) [47]	Mexico	609 athletes; age: 11–18 y (AM = 13.95 y); 18 sports.	Transversal	Study the perception of autonomy support given by the coach, the satisfaction of the BPNs, SWL, and SV; analyse the mediating role of the BPNs between the perception of autonomy support and the SWL and SV.	Support for autonomy; BPNs; SWL; SV.	BPNs are a positive and significant predictor of both SWL and SV; BPN-Aut satisfaction is the one that presents the highest prediction value for SWL and SV.	F
Mack et al., (2011) [51]	Canada	219 volleyball players (92 M; 127 F); age: 18–28 y (AM = 20.03 y).	Transversal	Study the relationships between BPN satisfaction, SV, PA, and NA.	BPNs; SV; PA; NA	Positive, significant, and moderate correlations between BPNs and SV and PA; negative and significant and moderate correlation between BPNs and NA; BPNs’ satisfaction were positive and significant predictors of SV and PA and negative predictors of NA.	F
Vansteenkiste et al., (2010) [54]	Belgium	Study 1: 304 football players; AM (24.66 y); Study 2: 245 athletes from 17 football clubs of various levels of the Belgian Nationa League; AM = 24.3 y.	Transversal	Examine whether autonomous and controlling reasons for adopting performance targets would provide more information on the relationship between performance goals and BE and fair-play’s attitudes and behaviours.	Performance goals; autonomous and controlled reasons; SV; PA and NA; pro- and antisocial behaviours; athletic attitudes.	The autonomous reasons were positive and significant predictors of SV and PA and negative, and not significant, predictors of NA; the controlled reasons have a negative and non-significant relationship with SV, negative and significant relationship with PA, and positive and significant relationship with NA.	G
Balaguer et al., (2008) [48]	Spain	301 athletes from various sports (171 M; 130 F); AM = 24.1 y.	Transversal	Analyse the relationships between the perception of support for autonomy provided by the coach, the BPNs, SMD, SE, and SWL.	Autonomy support; BPNs; SMD; SE; SWL.	BPNs were positive and significant predictor of SDM; and SDM was a positive and significant predictor of SE and SWL.	G
Solberg and Halvari (2009) [55]	Norge	95 elite athletes (56 M; 39 F); age: 14–44 y (AM = 21.6 y).	Transversal	Study the relationships between coach autonomy support, characteristics of personal goals, and EWB in elite athletes.	Perceived autonomy support; ARs; CRs; objective content (intrinsic and extrinsic); PA and NA.	Support autonomy was significant and positively related with PA; ARs were a positive and significant predictor of PA; CRs were a positive and significant predictor of negative PA.	G
Smith et al., (2007) [52]	United Kingdom	210 athletes (104 M, 103 F, 3 not specified); age: 18–37 y (AM = 21.02 y).	Mixed methodology	Analyse the motivational process underlying the sports goals, as well as the role of supporting the coach’s autonomy in relation to achieving the goals.	Personal goals; BPNs-Aut; BPNs-Comp; BPNs-Rel; autonomy support; PA and NA; SWL.	Moderate positive and significant correlations between the three BPNs, PA, and SWL; negative, significant, and weak correlation between the three BPNs and NA.	F
Gagné et al., (2003) [53]	EUA	33 gymnastics athletes (33 F); age: 7–18 y (AM = 13 y).	Longitudinal	Analysis of the effects of support perceptions provided by coaches and parents on BPN satisfaction, motivation, and WB.	BPNs; RAI; SV; SE; PA; NA; skill level in gymnastics.	Id Reg was a positive and significant predictor of PA, SV, and SE; Id Reg was a non-significant negative predictor of the NA; Int Mot was a positive and significant predictor of PA, SV, and SE; Int Mot was a negative and significant predictor of NA; RAI was a positive and significant predictor of the PA, SV, and SE; RAI is a negative and significant predictor of the NA.	G
Vallerand et al., (2006) [50]	Canada	Study 1: 206 (84 M; 119 F; 3 not specified). Age: 17–29 y (AM = 18.70 y). Study 2: 210 basketball players (129 M, 78 F; 3 not specified); age: 12–29 y (AM = 16.04 y). Study 3: 107 athletes (63 F; 44 M); age: 11–33 y (AM = 15.46 y); sports: 79 athletes of water polo and 28 of synchronised swimming.	Study 1: Transversal Study 2: Transversal Study 3: Longitudinal–Prospective	Study 1: Testing a sequence of experiments from the DMP to study the determinants of HP and OP. Study 2: Study the role of passion in the experience of affective variables in the context of sport. Study 3: Study the integrative sequence involving the determinants and affective experiences associated with PH and PO.	Study 1: HP and OP; personality orientation; sports rating. Study 2: HP and OP; SWL; PA and NA; SV; SWB. Study 3: PH and PO; personality orientation; SV; AP and AN; SWB.	Study 1: Sports evaluation and an autonomous personality orientation positively predict HP. Sports evaluation and controlled personality orientation positively predict OP. Study 2: HP showed positive, significant, and moderate correlations in absolute terms with PA, SV, and SWB;PH showed a negative, insignificant, and weak correlation with NA; OP showed positive, significant, and weak correlations with PA, NA, and SV; OP showed a positive, non-significant, and weak correlation, in absolute terms, with SWB. Study 3: PH showed a positive, significant, and weak correlation with WB. The PO is a positive, non-significant, and weak correlation with WB. PH was a positive and significant predictor of WB, while PO was a negative and non-significant prediction of WB.	G
Vallerand et al., (2008) [30]	Canada	Study 1: 184 basketball athletes (108 M; 76 F); AM = 16 y. Study 2: 67 athletes; (22 M; 45 F); age: 13–33 y (AM = 16.1); modalities: water polo and synchronised swimming.	Study 1: Prospective. Study 2: Prospective.	Study 1: This study aimed to determine if both HP and OP positively predict deliberate practice, which in turn positively predicts performance. Study 2: Examine the role of AGs as mediators of passion, their relationship with deliberate practice, and as predictors of sporting performance.	Study 1: HP and OP; Deliberate practice; Performance. Study 2: HP and OP; SWL; AGs; Deliberate practice; Performance; SWL	Study 1: HP is a predictor of deliberate practice, which in turn predicts performance (HP represents an important determinant of strong involvement in activities that athletes enjoy). Study 2: A positive, significant, and moderate correlation was found between HP and SWL. A positive, non-significant, and weak correlation was found between OP and SWL.	G

Note: M = Male; F = Female; AM = Age Mean; y = Years; PA = Positive Affect; NA = Negative Affect; SE = Self-Esteem; WB = Well-being; EWB = Emotional Well-being; PWB = Psychological Well-being; SWB = Subjective Well-being; SDM = Self-Determined Motivation; Amot = Amotivation; MI = Intrinsic Motivation; Reg Ext = External Regulation; Reg Id = Identified Regulation; BPNs = Basic Psychological Needs; BPNs-Comp = Basic Psychological Needs of Competence; BPN-Rel = Basic Psychological Needs of Relatedness; BPNs-Aut = Basic Psychological Needs of Autonomy; AGs = Achievement Goals; P = Passion; HP = Harmonious Passion; OP = Obsessive Passion; RAI = Global Self-Determination Index; ARs = Autonomous Reasons; CRs = Controlled Reasons; SWL = Life Satisfaction; SV = Subjective Vitality; DMP = Dualistic Model of Passion; SDT = Self-Determination Theory; G = Good; F = Fair.

## 4. Discussion

The present study aimed at systematically reviewing the relation between passion, behaviour regulation, and SWB in sport athletes. The main findings allowed us to identify that HP and autonomous motivation (i.e., more self-determined forms of behaviour regulation) are positively related to SWB, while OP and controlled motivation (i.e., less self-determined forms of behaviour regulation) are negatively associated with positive affect and SWL. It was also observed that BPNs mediated the relationship between HP and SWB but did not mediate the relationship between OP and SWB. It was also verified that BPNs have a positive impact on SWB.

These results corroborate the previous research [31,32,33,34,35] conducted with these variables in different areas. It means that in a competitive sport context, passion and behavioural regulation impact the SWB. Moreover, the importance of BPNs in SWB promotion in this field was found.

The DMP is based on the SDT assumptions, proposing that pleasant activities (those activities in which people like and engage in) are internalised in the identity of people, being highly valued by these people [20]. Moreover, when the internalisation of a pleasant activity occurs within the identity itself, it leads to a passion for that specific activity [43]. Hence, when people experience HP, they are likely to enjoy activities that strengthen their overall well-being and intrinsic motivation, making them more likely to continue engaging in the activity driven by autonomous regulation. It means that subjects feel free to choose to engage in the activities rather than feeling under pressure or forced [44]. On the other hand, OP is the driver when individuals internalise their actions into their individual identity under external pressure (e.g., social pressures) [19]. This type of passion often stems from external pressures or internal contingencies, such as the need for social acceptance, self-esteem, or other external rewards; as a result, their behaviour is controlled, meaning they feel less autonomy and more external or internal pressure to perform [44]. Moreover, OP is involved when the subjects cannot help themselves and surrender to their desire to engage in the passionate activity [44], and it also results from a controlled internalisation [46]. In this way, people who regulate motivation autonomously demonstrate greater positive affect and greater SWL, while people who regulate their behaviour according to controlled motivation are more likely to experience negative affect, reduce SWL, and psychological distress [15].

In fact, the research has confirmed that the DMP and SDT are predictors of athletes’ well-being (e.g., Felton and Jowett [46], Carpentier et al. [56]), showing that autonomous motivation is a positive predictor of SWB, both in the emotional and cognitive dimensions [48,57], and controlled motivation is a negative predictor of SWB [49,53].

According to Vallerand et al. [25], HP is associated with autonomous regulation, because the subject practices the sporting modality by free will due to the taste he has for the activity and not for questions of strengthening his personal identity, and, on the other hand, OP is linked to a controlling regulation because the subjects force the practice of the modality, seeking, through its realisation, feelings of social acceptance or of increased self-esteem, pressing himself internally to realise it: a situation that can influence well-being or, more concretely, the SWB.

The study of Vansteenkiste et al. [54] with football players, with an age mean of 24 years old, beyond analysing other variables of well-being, identified that, in fact, the autonomous reasons to practice are positively associated with positive affect, the SWB variable. On the other hand, the controlled reasons present a negative and significant relationship with negative affect. Also, Carpentier and Mageau [49] identified, with a sample of athletes with an age mean of 15 years from various modalities (i.e., synchronised swimming, football, athletics, and ice hockey), that autonomous motivation is positively related to SWB, while less self-determination motivation and more controlled behaviour are negatively associated with SWL and positive affect. Also, Gagné et al. [53], in their study with young gymnastics, observed that behavioural regulations linked with autonomous motivation (i.e., identified regulation; intrinsic motivation) were also positively and significantly linked to positive affect. This means that the athletes, when involved in sport practice according to autonomous regulation, independently of modalities or age, identify more positive affect, SWL, and SWB comparatively to those who are involved for controlled reasons. 

Regarding passion, it was also identified that HP has a positive effect on well-being. Verner-Filion et al. [28], through a sample of football players, identified that HP has a positive and significant effect on SWL, a cognitive variable of SWB. Vallerand et al. [50] confirmed, with basketball players, the positive influence of HP on SWL and positive affect, and that there is no significant relationship between OP and the SWB variables. This confirms, in a sports context, the positive effect of HP on SWB. Thus, the athletes who choose to engage freely in the sporting activities have a greater perception of positive affect and SWL in comparison to those who are under pressure or forced to it.

In this way, the retrieved studies for this review also highlight the importance of BPNs on these variables in a sports context, independently of age or modalities, since diverse studies with samples constituted by different ages and modalities tested the positive effect of BPNs on behavioural regulation and SWB. López-Walle et al. [47] conclude in their study with 609 young athletes from 18 sports that BPNs predict positively SWL. Mack et al. [51], through a sample constituted by 219 volleyball players with an age mean of 20 years old, found a significant and positive relationship between BPN satisfaction and positive affect and a negative association between BPNs and negative affect. Also, Smith et al. [52], in their study constituted by 210 adult athletes, verified the positive and significant influence of each BPN (i.e., autonomy, competence, and relatedness) on positive affect and SWL. In other strands, the study of Felton and Jowett [46] aspired to verify whether BPN satisfaction is a mechanism by which the binding style provided by parents or coaches is related to well-being. The results indicate that satisfaction with the BPNs provided by parents and coaches was a positive and significant predictor of positive affect and, at the same time, a negative and meaningful predictor of negative affect. These results also corroborate the findings of Solberg and Halvari [55], where the authors identified that, in elite sports athletes, support autonomy potentiates significantly positive affect, an emotional variable of SWB. Also, Balaguer et al. [48] identified that athletes’ perception of support autonomy by the coaches regarding BPN satisfaction positively predicted self-determination motivation, and it shows a positive association with self-esteem and SWL, the cognitive variable of SWB. This is very important and helps us to understand the motivational dynamic. Support autonomy means that an individual in a position of authority, like a coach over athletes, acknowledges their feelings and gives them opportunities for choice, while minimising the use of pressure and demands [57]. In this way, when subjects perceive their context as autonomy-supportive, they are more likely to experience the satisfaction of BPNs and more autonomous regulation of motivation, which is characterised by actions that are fully endorsed by the self and leads to well-being promotion [58]. In this way, autonomous motivation is essential for psychological growth, development, and well-being since people who engage in activities that satisfy their BPNs experience intrinsic motivation, which is related to SWB [58].

### 4.1. Practical Implications

Despite no retrieved study analysing the full sequence between the DMP, SDT, and SWB, we can highlight, through this review, the effect of each variable on SWB, demonstrating that HP, BPNs, and motivation regulation have a positive effect on the SWB of athletes in sports contexts. Therefore, based on this systematic review’s results, which support the assumption that HP and BPNs are essential to developing SWB in this sports context, it becomes essential to consider it in the promotion of athletes’ well-being.

To promote harmonious passion, according to Mageau et al. [59], environments should be fostered that support autonomy, athletes should choose acknowledging feelings, and rationales for task overrule should be provided. Identification with the activity also can contribute to fostering harmonious passion, since when individuals see an activity as part of their identity, they are more likely to value it and engage in it passionately. Regarding the child, parental influence also has an influence on HP, so it should be supportive rather than controlling, since parents who highly value a child’s activity and push for specialisation may inadvertently promote OP.

Regarding the results of the present study, which reinforce the influence of BPNs on the studied variables, it seems clear that in a sports context, the satisfaction of BPNs should be promoted. Hence, the BPN of autonomy should be provided by coaches offering choices. This promotes a sense of ownership about athletes’ activities and decisions (e.g., athletes choose their training schedules, goals, and strategies) [60]. Teaching athletes’ self-regulation techniques to manage their own training and performance goals helps to increase the BPN of autonomy [17]. For the BPN of competence, optimal challenges should be created, like setting goals and tasks that are challenging yet attainable, designed within the athletes’ skill levels, which promote the experience of success for them. Positive feedback that recognises effort and progress, emphasising mastery and improvement, should also be used as a strategy to promote the BPN of competence [60]. Regarding the BPN of relatedness, it is essential to promote supportive relationships between coaches, teammates, and athletes [17].

### 4.2. Study Limitations and Future Directions

Although we conducted an extensive search of the databases, we may have missed other studies. On the other hand, this review attempted to select a homogeneous study group by establishing detailed and complete inclusion criteria. However, even after a careful selection of studies, in addition to the scarcity, there was some diversity of methods, analyses, and results that may influence the findings in this study. Despite having tried to build a homogenous sample, the sample retrieved was heterogeneous. Some studies have samples with a wide range of ages and sporting modalities. Therefore, it is not possible to understand the results according to age, modality typology (i.e., individual or group), or gender. Hence, to improve the knowledge about this area and to specify the practical implications, we recommend for future reviews about on this topic an inclusion criterion that allows for building a more homogeneous analysis. 

The study goal of the present review was to analyse the effect of the DMP and behavioural regulation on SWB. However, well-being is a widely accepted concept that includes other dimensions and variables (e.g., subjective vitality; happiness), and this review did not verify the impact of these variables on general well-being. In this way, to create conditions to promote the greater well-being of athletes, it seems also important to promote the study of the impact of the DMP and behavioural regulation on other well-being variables.

Through this study, we also highlighted the impact of support autonomy on behavioural regulation and SWB. It is important that in future reviews, researchers include concepts to better understand the impact of support autonomy on SWB.

## 5. Conclusions

Even though the fact that no studies were found that simultaneously analyse the three constructs, it was possible to identify several relations between the study’s variables.

In this way, through the main findings of this work, we can conclude that in the sports context, HP is positively linked to SWL and positive affect, and OP is negatively related to the same variables. Autonomous regulation of behaviour is a positive predictor of SWL and positive affect, and controlled regulation of behaviour is a negative prediction of the same variables. The results of this review also conclude that BPN satisfaction is positively related to SWL and positive affect and negatively related to negative affect. 

These results show us the importance of developing conditions in sport contexts that promote positive feelings that consider athlete’s development of harmonious passion and self-determined behaviour regulation, in order to achieve higher levels of well-being. Nevertheless, these results also lead us to the necessity for more studies to highlight the theoretical link between passion and motivation in the context of sport, especially regarding the mediating role of motivation in the relationship between passion and well-being. 

## Data Availability

Data are available under request to the first author.
